# Ultrafast hot electron dynamics in plasmonic nanostructures: experiments, modelling, design

**DOI:** 10.1515/nanoph-2022-0592

**Published:** 2023-01-12

**Authors:** Andrea Schirato, Margherita Maiuri, Giulio Cerullo, Giuseppe Della Valle

**Affiliations:** Dipartimento di Fisica – Politecnico di Milano, Piazza Leonardo da Vinci, 32, 20133 Milan, Italy; Istituto Italiano di Tecnologia, Via Morego 30, 16163, Genova, Italy; Istituto di Fotonica e Nanotecnologie – Consiglio Nazionale delle Ricerche, Piazza Leonardo da Vinci, 32, 20133 Milan, Italy; Istituto Nazionale di Fisica Nucleare, Sezione di Milano, Via Celoria, 16, 20133 Milan, Italy

**Keywords:** electron–phonon coupling, hot electrons, localized surface plasmon resonances, metal nanoparticles, ultrafast spectroscopy

## Abstract

Metallic nanostructures exhibit localized surface plasmons (LSPs), which offer unprecedented opportunities for advanced photonic materials and devices. Following resonant photoexcitation, LSPs quickly dephase, giving rise to a distribution of energetic ‘hot’ electrons in the metal. These out-of-equilibrium carriers undergo ultrafast internal relaxation processes, nowadays pivotal in a variety of applications, from photodetection and sensing to the driving of photochemical reactions and ultrafast all-optical modulation of light. Despite the intense research activity, exploitation of hot carriers for real-world nanophotonic devices remains extremely challenging. This is due to the complexity inherent to hot carrier relaxation phenomena at the nanoscale, involving short-lived out-of-equilibrium electronic states over a very broad range of energies, in interaction with thermal electronic and phononic baths. These issues call for a comprehensive understanding of ultrafast hot electron dynamics in plasmonic nanostructures. This paper aims to review our contribution to the field: starting from the fundamental physics of plasmonic nanostructures, we first describe the experimental techniques used to probe hot electrons; we then introduce a numerical model of ultrafast nanoscale relaxation processes, and present examples in which experiments and modelling are combined, with the aim of designing novel optical functionalities enabled by ultrafast hot-electron dynamics.

## 1 Introduction

The unique optical properties of plasmonic nanostructures, which find no counterpart in bulk materials, are intimately linked to the spatial confinement of free electrons in metals down to the nanoscale [1]. Light interacts strongly with the free conduction-band electrons of metallic (typically Au, Ag, Cu) nanoparticles (NPs), resonantly exciting localized surface plasmons (LSPs), i.e. collective charge oscillations coupled to the electromagnetic modes bound to the conductor/dielectric interface [2, 3]. Intuitively, the external field induces charges on the surface of the metal, which promote a restoring force that drives further electric currents, sustaining the electron oscillation [4, 5]. The phenomenon is known as LSP resonance (LSPR), and enables a high light mode confinement in nanometric volumes [6, 7]. For this reason, plasmonic NPs have been extensively studied in the last decades and exploited in a variety of contexts benefiting from the enhanced coupling with light [8–13], such as photodetection [14–16], solar energy harvesting [17–19], subwavelength nonlinear optics [20–24], and sensing [25–29].

LSPs dephase very quickly and, by decaying, release their energy to create electron–hole pairs with very high energies [30–33]. These carriers are referred to as ‘hot’, namely in a non-equilibrium state with much higher energy than at the thermodynamical equilibrium. In their higher-energy states, hot electrons and holes promote multiple processes otherwise unattainable [34–37]. Driving photochemical reactions at the metal surface to increase catalytic yields [38–45], collecting carriers to enhance photodetection and photovoltaics [46–53], or modulating light with unprecedented speed [54–59] are only few of the intriguing applications explored so far. Yet, such opportunities are limited by the ultrafast internal relaxation of carriers, which, following photoexcitation, dissipate the absorbed light energy by equilibrating with the environment and converting it into heat [60–64]. This process promotes an ultrafast (picosecond timescale) temperature increase of the metal NP and the surrounding environment, providing a local heating mechanism via efficient photothermal transduction [65–72]. This localized heating can be exploited for several applications such as, in combination with suitable surface functionalization, plasmonic photothermal therapy of tumors [73–76]. Due to their ability to combine light capture and energy conversion at the nanoscale, hot electrons in plasmonic nanostructures have recently attracted increasing attention and opened novel routes in science and applications [77].

The promising advances in plasmonic hot carrier physics and technology rely on the understanding of the nonequilibrium mechanisms which regulate the interaction between light, the electrons in the metal and the environment of the nanostructure. Towards this direction, a considerable contribution has come from transient absorption (TA) spectroscopy, rapidly advancing the field of ultrafast plasmonics [78–80]. Femtosecond pump-probe experiments have become a powerful tool to unveil the energy exchange processes governing hot carrier equilibration, by allowing to track their evolution in time [81–99]. However, to interpret the results and understand the wealth of phenomena involved in plasmonic hot carrier photogeneration and relaxation, it is crucial to combine time-resolved experimental studies with suitable modelling tools. The development of accurate models would indeed enable the design of nanodevices implementing hot-carrier-driven advanced functionalities: being able to provide quantitative predictions of the optical response of such systems would allow one to engineer and control the hot carrier photophysics, in view of their applications.

Modelling photoexcited electrons in plasmonic nanostructures is, however, far from straightforward. Indeed, while the LSP excitation is adequately accounted for by classical electromagnetism [1], the dynamics of hot carriers [100–105] and their impact on the optical properties of the metal [106–108] necessitate more sophisticated theories. The challenge is made even more arduous by the variety of parameters, such as the nanostructure material and environment, its geometry and size, as well as the electronic band structure and the energies of the involved photons, plasmons and carriers. Although a holistic framework for these ingredients would be desirable, a model relying on a single level of theory to comprise all the mechanisms seems unrealistic. Several approaches have therefore been proposed, with different degrees of detail of the substantially mismatched length, energy and time scales concerned. However, in order to practically design nanostructures for a specific ultrafast functionality, models should be at the same time accurate and computationally handy.

This twofold requirement fosters the development of a reduced model that, at the expense of the accuracy of a first-principles description of the electronic population, could capture the essential physical mechanisms dictating the hot carrier dynamics and the nonlinear modification they induce on the optical response of the plasmonic nanostructure. Ideally, such a modelling approach should be: (i) as simple as possible, relying on a limited number of degrees of freedom; (ii) easy to apply in a variety of nanostructure configurations (including small nano-objects in the quasi-static limit, larger nanoantennas exhibiting a retardation-based response, colloidal nanoparticle ensembles, extended periodic systems as metasurfaces); (iii) validated by experimental observations. Upon these conditions, the model could be applied to the design of novel structures and optimized solutions, exploiting the ultrafast light–matter interaction mediated by hot carriers.

With this review, we aim to show that such a combination of experiments, modelling and design is indeed possible. To do so, we report some of our recent efforts to investigate the dynamics of hot carriers in various plasmonic structures, where experimental and numerical results are systematically combined and compared. This enables us to validate our modelling approach, which balances accuracy and flexibility, and to identify some design principles directly tested by our experiments. It is not our intent to provide an exhaustive review of the vast plasmonic hot carrier literature, for which we address the readers to more focused publications (e.g. refs. [109–116], to mention a few). Here we rather present the basics of our experimental and theoretical approaches, which might be useful for the design of future plasmonic nanodevices. For review on other aspects of plasmonic hot carriers, see e.g. refs. [117–128].

This review is organized as follows. In Section 2 we present the general concepts of plasmonics and hot carrier dynamics. Section 3 provides an overview of the experimental techniques used to track the relaxation of hot electrons in various metallic nanostructures. In Section 4 we discuss common theoretical approaches to describe carrier relaxation and present our own numerical modelling technique. The most representative results of experiments and simulations on the ultrafast dynamics of hot electrons, alongside examples of designed nanostructures to achieve advanced optical functionalities are reported in Section 5. Section 6 concludes the review and draws perspectives for future works.

## 2 Fundamental concepts of plasmonics and hot carrier physics

**Figure 1:**
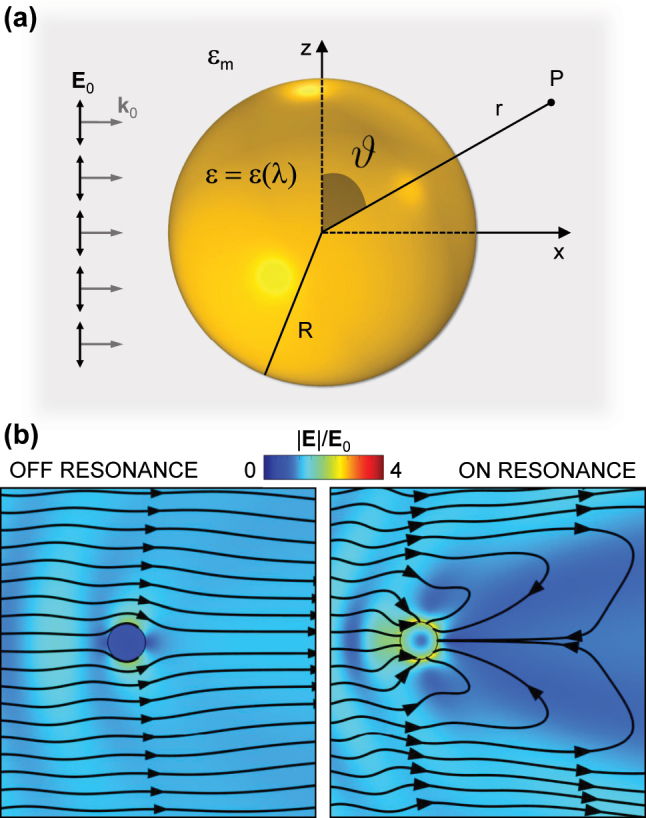
Localized surface plasmon resonance in small NPs. (a) Sketch of a metal spherical NP of radius *R* and permittivity *ɛ*, embedded in a homogeneous dielectric medium of permittivity *ɛ*_m_ and subjected to an electrostatic field of amplitude *E*_0_ linearly polarized along the *z*-axis. (b) Electric field enhancement (colour map) and field lines of the total Poynting vector (excluding that scattered) around a small (*R* = 60 nm) Ag NP in air, off resonance (left, *λ* = 600 nm) or on resonance (right, *λ* = 346 nm).

Classical electromagnetism can be employed to capture the most relevant aspects of coupling light from free space to surface plasmons in the linear regime, as briefly discussed in the following. More specifically, we will describe the properties of LSPs in the limit of extremely small nanostructures, starting from the simplest case of a spherical NP with radius *R*. Provided that *R* is much smaller than the wavelength *λ* of the incoming radiation, it is possible to study the effects of photoexcitation within the so-called quasi-static approximation (QSA), which assumes that excitations do not propagate inside the metal and neglects retardation effects across the NP [1, 129]. In this framework, we consider a NP with a wavelength-dependent complex-valued permittivity *ɛ* = *ɛ*(*λ*), embedded in a homogeneous and isotropic dielectric medium of constant permittivity *ɛ*_m_ and excited by a monochromatic electric field, propagating along the *x*-axis and linearly polarized along the *z*-axis, $E_{0}=E_{0}{\text{e}}^{\text{i}\left(k_{0}x-\omega t\right)}u_{z}$ (see Figure 1a). Within the QSA, the problem of light scattering from the metal NP is formally equivalent to an electrostatic problem (electric and magnetic phenomena are decoupled, and the phase of the electric field is constant across the NP) [129], hence its solution is given in terms of an electrostatic potential Φ. Specifically, the potential outside the nanosphere Φ_out_ can be obtained by solving Laplace equation ∇^2^Φ = 0 with suitable continuity boundary conditions, and written [1, 3]:

(1) $$\Phi_{\text{out}}=-E_{0}r\;cos\;\vartheta +\frac{\varepsilon -\varepsilon_{\text{m}}}{\varepsilon +2\varepsilon_{\text{m}}}\frac{R^{3}}{r^{2}}E_{0}\;cos\;\vartheta ,$$

where *ϑ* is the polar angle between the position vector *r* at point *P* and the *z*-axis (Figure 1a). Note that Φ_out_ is the superposition of two contributions: the first is the potential corresponding to the incident electric field **E**_0_, while the second term can be interpreted as the potential generated by a point-like electric dipole **p**, located at the center of the NP and induced by **E**_0_. The expression of **p** is given by:

(2) $$p=4\pi \varepsilon_{0}\varepsilon_{\text{m}}R^{3}\frac{\varepsilon -\varepsilon_{\text{m}}}{\varepsilon +2\varepsilon_{\text{m}}}E_{0},$$

with *ɛ*_0_ the vacuum permittivity, and corresponds to a complex-valued polarizability

(3) $$\alpha =4\pi \varepsilon_{0}R^{3}\frac{\varepsilon -\varepsilon_{\text{m}}}{\varepsilon +2\varepsilon_{\text{m}}}.$$

One can see that such polarizability displays a resonance (the LSPR, centred at *λ*_LSPR_) when |*ɛ* + 2*ɛ*_m_| is minimum. Under the approximation of small or weakly dispersed $\text{Im}\left\{\varepsilon \right\}$ , this condition simplifies to the so-called *Fröhlich resonance* condition:

(4) $$\text{Re}\left\{\varepsilon \left(\lambda_{\text{LSPR}}\right)\right\}=-2\varepsilon_{\text{m}}.$$

Starting from the NP polarizability, straightforward calculations lead to the expression of the total power absorbed (*P*_abs_) and scattered (*P*_sca_, i.e. re-radiated) by the NP, for a given intensity *I*_0_ of the incident plane wave [129]. The corresponding absorption and scattering cross-sections can then be defined as *σ*_abs_ = *P*_abs_/*I*_0_ and *σ*_sca_ = *P*_sca_/*I*_0_, respectively, with extinction cross-section being calculated as the sum of the two, *σ*_ext_ = *σ*_abs_ + *σ*_sca_. The resonant behaviour of *α* is thus inherited by the cross-sections of the NP, which dissipates the LSP mode energy much more efficiently when the resonance is matched, either into heat via Joule effect (absorption), or into radiation due to charge oscillations (scattering). To visualize the interaction of an electromagnetic field with a NP, Figure 1b exemplifies the optical behaviour of a small (60-nm radius) plasmonic NP (made of silver in air) excited by a monochromatic plane wave (linearly-polarized along the *z*-axis) either off resonance (left) or at the Fröhlich condition (right). The colour map refers to the field enhancement, higher and spatially localized at the NP surface on resonance, while the field lines of the Poynting vector are represented in black, showing a strong convergence near the NP when matching the resonance. By interpreting the effect in terms of cross-sections, the lines redirected onto the particle define an absorption radius [130] much greater than the geometrical one, and the NP presents a larger target to the incoming radiation. On the other hand, at off-resonance wavelengths, light propagation is weakly perturbed by the NP.

**Figure 2:**
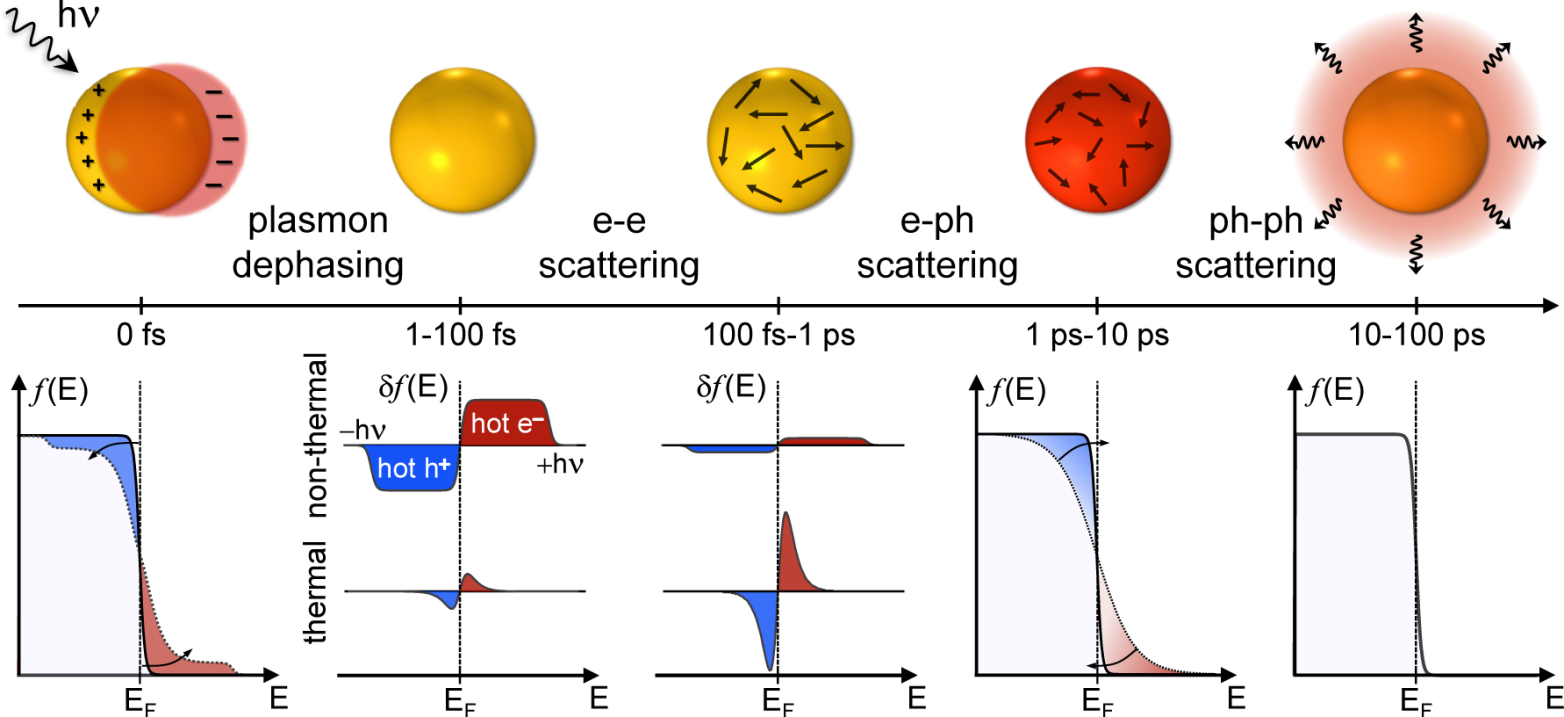
Photoinduced relaxation processes in a plasmonic nanostructure. Schematic representation of the relaxation processes following photoexcitation of a plasmonic NP. The main energy exchange mechanisms and characteristic time scales involved are indicated. The electronic distribution *f*(*E*) is also depicted, highlighting the non-thermal and thermal hot electrons (red) and holes (blue) contributions around the Fermi energy *E*_*F*_.

When dealing with the temporal dynamics of a NP following LSP excitation, it is worth highlighting that the driven oscillation of free electrons in the metal loses its coherence very quickly: within ∼10 fs the plasmon decays either radiatively (by emitting a photon) [15, 131], or non-radiatively, into single-particle electronic excitations (Landau damping) [132, 133]. Interestingly, the non-radiative decay initiates a cascade of ultrafast photophysical processes, schematically depicted in Figure 2 alongside their characteristic time scales and carrier energy distributions [110]. Dephasing of the LSP first results in the generation of hot electrons (holes), promoted to states well above (below) the Fermi level *E*_*F*_. The ensuing energy distribution *f*(*E*) deviates from a Fermi–Dirac-like occupation probability and is not associated with an electronic temperature. The corresponding variation in the electronic energy distribution *δf*(*E*) is dominated by a faster non-thermal contribution from carriers with energies *E* spreading up to that of the impinging photon *hν*. Subsequently, within the first hundred femtoseconds, hot carriers undergo internal non-radiative relaxation processes, evolving from a non-thermal to a thermal energy distribution via electron–electron (e-e) scattering.

Thus, the corresponding *δf*(*E*) acquires a stronger thermal character and is peaked around *E*_*F*_. Then, within a few picoseconds, the excited carriers equilibrate with the metal phonon bath via electron–phonon (e-ph) scattering events, which gradually bring the electronic distribution back to equilibrium and transfer the absorbed photon energy towards the metal lattice. This mechanism promotes a local increase of the plasmonic NP temperature. Finally, via phonon–phonon scattering on much longer time scales, mainly from hundreds of picoseconds to nanoseconds until the stationary regime, heat is released from the NP towards its surrounding environment, which in turn experiences a temperature increase localized around the nanostructure.

The current understanding of the hot-carrier relaxation mechanisms outlined in Figure 2 relies on the combination of an extensive set of experimental and theoretical investigations. In the following sections, we will report on some of the techniques currently employed to track the ultrafast dynamics of the energy flow regulated by the short-lived photoexcited states of the hot carriers.

## 3 Experimental techniques for studying hot electron dynamics in plasmonic nanostructures

**Figure 3:**
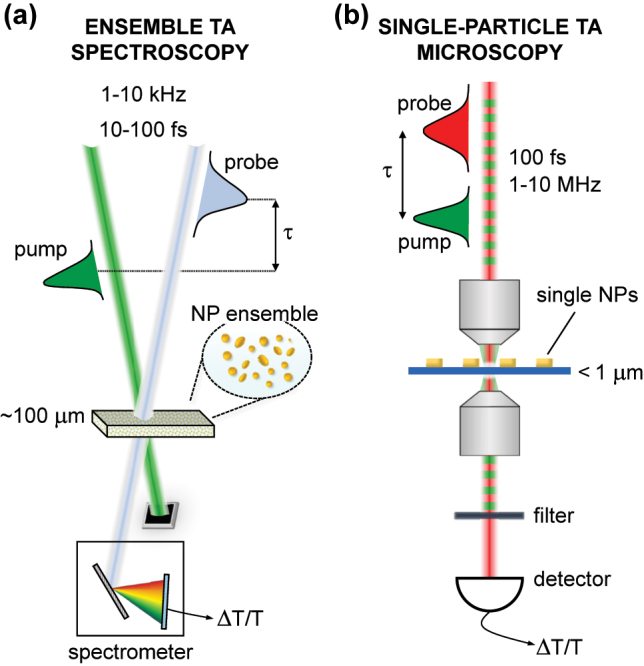
Experimental techniques for studying hot carrier dynamics in plasmonic nanostructures. (a) Sketch of a TA spectroscopy setup to study an ensemble of nanostructures. (b) Sketch of a TA microscopy setup to perform single-particle measurements.

Femtosecond TA spectroscopy [80] is a very powerful technique for tracking photoinduced energy flow in plasmonic nanostructures. Figure 3 shows two TA spectroscopy schemes, employed either for more conventional ensemble measurements (Figure 3a), or in the limit of single-particle detection in a TA microscopy configuration (Figure 3b). In the next paragraphs, we describe more in detail the two apparatuses.

### 3.1 Ensemble transient absorption spectroscopy

Femtosecond TA spectroscopy is particularly simple when applied to ensembles of nanostructures, either dispersed in solution or deposited on a substrate. A basic experimental setup for broadband femtosecond TA spectroscopy is shown in Figure 3a. The TA setup is driven by energetic femtosecond laser pulses, typically generated by an amplified Ti:sapphire laser system working at 1–10 kHz repetition rate and producing ∼100-fs pulses at 800 nm with energy >1 mJ. A fraction of the laser output is used to generate the pump pulses, either by second harmonic generation (SHG) to 400 nm or by pumping an optical parametric amplifier (OPA) producing tunable visible (450–750 nm) or near-infrared (1.1–2.7 μm) pulses [134]. Typically, OPAs generate pulses with a ∼100-fs duration, comparable to that of the driving pulses; however, non-collinear OPAs (NOPAs), combined with suitable dispersion compensation techniques, can generate significantly shorter pulses, with a duration of 10–15 fs, tunable both in the visible (500–700 nm) and in the near-infrared (820–2000 nm). A second small fraction of the laser output, with energy of the order of a few μJ, is used to produce the probe pulses, typically by white-light continuum (WLC) generation in a thin (1–4 mm) plate of a dielectric material. Different materials can be employed to generate the WLC, depending on the wavelength region of interest. Sapphire is used for the 450–750 nm range, while CaF_2_ allows to extend the spectral coverage to the ultraviolet, down to 320 nm, and YAG is optimum for the near-infrared range (820–1500 nm) [135]. One of the advantages of the WLC is the remarkable energy stability, with typical shot to shot energy fluctuations which can be as low as 1 − 2 × 10^−3^ for most wavelengths of the probe spectrum.

Pump and probe pulses are synchronized and their delay *τ* is varied by a computer-controlled mechanical delay line. Pump and probe pulses are non-collinearly focused into the sample, in which they are spatially overlapped, down to diameters of the order of 100 μm; the transmitted probe pulse, spatially selected by an iris, is sent to a spectrometer, with the capability to record probe spectra for each laser shot. By measuring the transmitted probe spectrum in the presence of the pump, *I*_ON_(*λ*, *τ*) and the corresponding spectrum in the absence of the pump (which does not depend on the delay), *I*_OFF_(*λ*) and by defining the sample transmission: *T*_ON(OFF)_ = *I*_ON(OFF)_/*I*_0_ with *I*_0_(*λ*) the incident probe spectrum, one can finally calculate the differential transmission (Δ*T*/*T*) spectrum as:

(5) $$\begin{array}{ll} \frac{\Delta T}{T}\left(\lambda ,\tau \right) & =\frac{T_{\text{ON}}-T_{\text{OFF}}}{T_{\text{OFF}}}\\ & =\frac{I_{\text{ON}}\left(\lambda ,\tau \right)/I_{0}\left(\lambda \right)-I_{\text{OFF}}\left(\lambda \right)/I_{0}\left(\lambda \right)}{I_{\text{OFF}}\left(\lambda \right)/I_{0}\left(\lambda \right)}\\ & =\frac{I_{\text{ON}}\left(\lambda ,\tau \right)}{I_{\text{OFF}}\left(\lambda \right)}-1. \end{array}$$

TA spectroscopy therefore measures a 2D map representing the Δ*T*/*T* signal as a function of probe wavelength *λ* and pump-probe delay *τ*. Due to propagation in the material used for WLC generation and in other optical elements in the beam path, the probe pulse acquires a frequency chirp. This results in a probe wavelength dependence of the zero pump-probe time delay (*τ* = 0) which can be corrected by a suitable calibration (the so-called ‘de-chirping’ procedure). Once this is done, it can be shown that the temporal resolution of the TA signal is not dependent on the chirp of the probe pulse but is only determined by the pump-pulse duration [136]. It is therefore possible to measure TA signals with a time resolution ranging from ∼100 fs when using OPA pump pulses down to ∼10 fs when employing a NOPA.

The sensitivity of broadband TA spectroscopy can be obtained by considering typical shot to shot fluctuations of 2 × 10^−3^ between consecutive probe pulses. When averaging over 400 consecutive probe pulse pairs which, for kHz repetition rates, correspond to an acquisition time of the order of 1 s, one obtains a fluctuation of 10^−4^, which can then be reduced to a few times 10^−5^ with further averaging. One can therefore measure with high signal-to-noise ratio (SNR) Δ*T*/*T* signals larger than 10^−4^, which are typically achieved with ensemble measurements. To measure smaller signals, as those generated by single NPs, one needs to increase the sensitivity by using laser systems with higher repetition rate, which will be described in the next paragraph.

### 3.2 Single-particle transient absorption microscopy

TA experiments are typically performed on large ensembles of NPs, of the order of 10^8^ - 10^12^ in a typical focal volume (∼100 μm^3^). The pump pulse excites and mutually synchronizes a subset of such NPs, whose time evolution is tracked by the delayed probe pulse. The implicit assumption of such ensemble experiments is that all the interrogated NPs are identical and evolve in the same fashion. For many systems this is not the case, since the NPs, even if they are all nominally identical, inevitably present a distribution of internal (size, shape, defects, etc.) as well as external (environment, interface with other nano-objects, etc.) characteristics. Standard TA experiments therefore measure dynamics which are averaged over large heterogeneous ensembles: while still providing very useful information, they often obscure the elementary dynamical processes that occur at the level of a single NP.

To measure the TA signal of a single NP, one has to face the experimental challenge of a much lower Δ*T*/*T* signal. The absorption cross-section of a metal NP, in fact, can vary in the range *σ*_abs_ = 10^−14^ − 10^−13^ cm^2^, depending on its size and shape. The expected maximum TA signal from a single nanoparticle can be expressed as:

(6) $${\left(\Delta T/T\right)}_{\max}=\xi \;\sigma_{\text{abs}}/S,$$

where *ξ* = 0.01 − 1 is a numerical factor typically smaller than one (unless plasmonic NPs at resonance are considered) and *S* is the cross section of the probe beam. For a focal beam diameter of the order of 100 μm, one would get *S* ∼ 10^−4^ cm^2^ and thus, assuming *ξ* = 0.1, (Δ*T*/*T*)_max_ = 10^−11^ − 10^−10^, which is well below the sensitivity of ensemble TA spectroscopy. One therefore needs to focus the probe pulse as tightly as possible. Using a diffraction-limited focus down to a diameter of 300–400 nm, one gets *S* = 10^−9^ cm^2^ so that (Δ*T*/*T*)_max_ = 10^−6^ − 10^−5^. Such levels of TA are typically too low for the kHz frequency lasers described in the previous paragraph, and require laser systems with higher repetition rate, which guarantee higher sensitivity.

The previous discussion highlights the two key requirements for performing TA experiments on single NPs: (i) a microscopy configuration, in order to focus the pump and probe beams down to diffraction-limited diameters of the order of ∼*λ*/2; (ii) a high repetition rate laser system, to enhance the sensitivity of the measurement by increasing the number of averages for a given measurement time. Figure 3b shows the typical experimental setup of an ultrafast TA microscope. It starts with a high repetition rate laser system, such as a mode-locked Ti:sapphire oscillator, working at *f*_rep_ = 100 MHz. The laser output is split into two beams, one for the pump line and the other for the probe line. The pump beam (following SHG in some cases) is sent to a high-speed modulator, typically an acousto-optic modulator (AOM), working at modulation frequencies of 1–10 MHz. The probe beam is sent to a nonlinear frequency conversion system, which can be either an optical parametric oscillator (OPO) or a nonlinear optical fiber, such as a photonic crystal fiber (PCF), for spectral broadening. Pump and probe pulses, synchronized by a mechanical delay line and collinearly recombined by a dichroic beam splitter, are sent to a high numerical aperture (NA) microscope objective (NA = 1 – 1.4) which focuses them onto the sample. The metal NPs are positioned on a microscope slide with a density sufficiently low that only one NP falls within the focused beam spot. Typically, a 3D nanopositioner allows to finely align the NP on the focused spot. The transmitted probe beam, recollimated by a second high-NA microscope objective and spectrally separated from the pump by a series of suitable filters, is then sent to a photodiode whose output signal is synchronously demodulated by a high frequency lock-in amplifier, sensitively extracting the pump-induced variation of the probe signal and, after suitable normalization, the Δ*T*/*T* signal. Note that this strictly collinear configuration requires the pump and probe beams to be at different frequencies, to enable spectral rejection of the transmitted pump light.

The use of a high repetition rate laser combined with high-frequency modulation and synchronous detection enables one to greatly increase the sensitivity of TA experiments. Laser sources are typically much quieter at high frequencies, moving away from the so-called 1/*f* noise and approaching the ultimate shot-noise limit. Considering a typical relative intensity noise (RIN) between −130 and −140 dB/Hz in the 1–10 MHz frequency range for a solid-state laser, one derives sensitivities in the range of 10^−7^ − 10^−6^ for integration times of the order of 1 s, which are sufficient to detect the Δ*T*/*T* signal of single NPs. TA microscopy thus typically works at a single wavelength and TA spectra can be collected by scanning the probe wavelength. Tunability of the probe pulse can be achieved by using an OPO pumped by the laser oscillator or by spectral broadening in a PCF followed by band-pass filtering.

## 4 Modelling the hot electron dynamics

The most rigorous description of the nonequilibrium processes in metal NPs involving hot carriers relies on first-principles calculations based on time-dependent density functional theory (TD-DFT) [137–139], which retain the full electronic band structure of the bulk material. The carrier density of states and matrix elements for optical transitions are derived without free parameters directly from the details of the full electronic structure, for an accurate resolution of the momentum and energy distributions of the photogenerated electrons and holes. This level of theory is particularly suited to study effects sensitive to the specific structure of electronic bands [140], as e.g. (i) photoinduced direct transitions, because of the precise selection rule between their initial and final states; (ii) the plasmon dispersion and anisotropy at the metal surface [141]; (iii) the initial anisotropic distribution of carrier momenta [30]. As an example, *ab initio* calculations of this kind have been employed to evaluate the energy-dependent impact of resistive losses, direct, and phonon-assisted transitions and to predict the carrier lifetimes and mean free paths as a function of the electron energies [142, 143]. TD-DFT also represents a unique tool to investigate the photoinduced charge transfer mechanisms taking place at the plasmonic NP surface [144, 145]. First-principles techniques have for instance been employed to corroborate experimental observations of a ballistic thermal injection at a metal/semiconductor interface [146], to rationalize ultrafast electron transfer dynamics in heterostructures [147] and 2D materials [148], or to gain insight into the effects of plasmons in photocatalysis [149].

Unfortunately, these calculations suffer from an applicability upper limit, set by numerical complexity. Computations can explicitly account for the nanostructure shape [150–152], but TD-DFT is suited to treat up to a few hundred atoms, i.e. systems up to 1–2 nm in size. Moreover, following their plasmon-assisted photogeneration, hot carriers undergo multiple scattering events, governing their spatio-temporal transport across the material. This additional phenomenon needs to be coupled to the carrier generation process in order to develop consistent models, for e.g. the extraction and collection of these high-energy electrons.

Among the available first-principles methods, the time-dependent Boltzmann equation (BE) offers a comprehensive theoretical framework [153, 154]. By means of the BE, one can study the dynamics of the out-of-equilibrium carriers by determining the evolution over time and (real) space of the electron energy and momentum distributions. This is done by tracking occupancy probability distributions in a six-dimensional phase space of spatial and momentum degrees of freedom, where energy-dependent scattering processes modify the state occupation. A nonlinear integrodifferential equation in six dimensions is thus obtained, with appropriate collision integrals to account for multiple scattering events and a source term coupling the system to an external exciting field. Such a powerful tool, however, soon becomes rather computationally demanding in its most general formulation.

Hence, despite the unparalleled insight and deep degree of accuracy offered by *ab initio* models, such approaches are unfeasible for nanostructures larger than ∼10 nm in size, which necessitate more versatile calculations. As a first step towards a reduction in complexity, one can simplify the electronic band structure to a free-electron one. Upon this approximation, analytical free-electron solutions and jellium TD-DFT can be employed in lieu of full band calculations [155–159]. At the expense of the atomic-scale description of the nuclear potential, similar approaches can be extended to complex nanostructures up to tens of nm in size, a crucial advantage to investigate geometry-assisted transitions and effects of the shape [31, 101, 160]. On the other hand, these approximated techniques fail as soon as materials and plasmon energies do not involve solely the free-electron-like conduction band of the metal. In parallel, simplifications of the time-dependent BE have also been proposed. Conventional approaches consider e.g. to restrict the space of the allowed distribution, to work in the steady-state regime and/or to neglect spatial diffusion, to simplify the collision integrals using the relaxation-time approximation [161–165]. Alternative hybrid computational solutions, derived from the BE, have been recently reported, and shown to capture the most relevant features of diffusive transport [166].

In spite of such approximations, the theoretical description of the relaxation mechanisms involving hot carriers remains, most generally, relatively complex. Furthermore, it is often not straightforward to integrate these models in multiscale and/or multiphysics solvers, coupling the carrier dynamics to other physical phenomena. Beyond the sophisticated techniques briefly outlined so far, a fundamentally different and more agile approach is the so-called two-temperature model (2TM) [167–170]. The 2TM relies on a substantially simpler formalism, based on thermodynamics. The dynamics of photoexcited electrons is treated as the thermalization process of two coupled thermal reservoirs, for the electron and phonon populations respectively. The process is formally described by a set of two coupled first-order differential equations, where a source term models the coupling with light. The electronic temperature first increases driven by photoexcitation, and then tends to equilibrate with the phononic thermal bath, which results in a cooling of the electron gas and an associated lattice temperature rise. Despite its remarkable simplicity, the 2TM, which can be formally derived from the BE [170], involves coefficients that can be calculated *ab initio* [171, 172] and has been employed to model a very large number of ultrafast experiments [173, 174]. The accuracy of the 2TM, however, is fundamentally limited by a strong assumption: electrons thermalize instantaneously, i.e. upon photoexcitation they populate electronic bands according to a Fermi–Dirac occupancy distribution, fully determined by the effective electron temperature. This makes the 2TM intrinsically inadequate to model the onset and early stages of the carrier dynamics, which are rather characterized by a non-thermal distribution and governed by e-e scattering events. In passing, we note that an equivalent hypothesis is made for phonons, assumed to be at thermal equilibrium throughout the entire dynamics. While this condition is generally fulfilled in noble metals, accounting for non-thermal phononic states may be relevant in other contexts and requires models beyond the 2TM [173].

For these reasons, the 2TM, while being extremely easy to handle, turns out to be too rudimentary when aiming at modelling the ultrafast dynamics of hot carriers. To relax the assumption on the instantaneous electron thermalization with a moderate increase in complexity, an extension of the model has been proposed, that we will detail in the following and suggest as an agile yet reliable tool to model the ultrafast hot carrier dynamics.

### 4.1 The three-temperature model (3TM)

The three-temperature model (3TM) is a semiclassical rate-equation model proposed for the first time on Au thin films [175], effectively balancing accuracy and computational complexity. To date, its use is well-established, and its predictions have been shown to reproduce experimental observations in a large variety of contexts.

Going beyond the shortcomings of the 2TM, the 3TM accounts for the photogeneration and ultrafast relaxation of hot carriers in terms of three internal energetic degrees of freedom for the plasmonic system.

Specifically, the model assumes the instantaneous dephasing of the LSP (Figure 2), with the excitation pulse that directly promotes a fraction of free electrons to a non-thermalized energetic distribution. The excess energy density stored in this population of out-of-equilibrium carriers, referred to as *N*, is the first energetic variable of the 3TM, which directly couples to the optical excitation. Such excess energy is subsequently released, via e-e scattering events [60, 95], towards a thermalized population of hot electrons, associated to an electronic temperature Θ_*E*_ higher than the room temperature Θ_0_. The energy (*E*) distribution of these hot carriers is a Fermi–Dirac-like function, $f\left(E,\Theta_{E}\right)={\left[1+e^{\left(E-E_{F}\right)/k_{B}\Theta_{E}}\right]}^{-1}$ , with *E*_*F*_ the metal Fermi energy and *k*_*B*_ the Boltzmann constant. Carriers tend then to equilibrate with the metal phonon bath, and e-ph scattering processes regulate the electron gas cooling and corresponding increase of lattice temperature, Θ_*L*_, until equilibrium is reached [176, 177]. Thus, the 3TM accounts for the relaxation mechanisms triggered by photoabsorption by interlinking *N*, Θ_*E*_ and Θ_*L*_ in a set of coupled rate equations which, once integrated over time, provide the ultrafast dynamical evolution of the hot carrier population. Note that, by explicitly including an energetic variable (*N*) to quantify the energy stored in non-thermalized electrons, the 3TM is among the simplest approaches to model photoexcitation of high-energy carriers without assuming their instantaneous thermalization [169].

More precisely, the 3TM reads as the following set of coupled ordinary differential equations [175]:

(7) $$\frac{dN}{dt}=P_{\text{abs}}\left(t\right)-aN-bN$$

(8) $$C_{E}\frac{d\Theta_{E}}{dt}=aN-G\left(\Theta_{E}-\Theta_{L}\right)$$

(9) $$C_{L}\frac{d\Theta_{L}}{dt}=bN+G\left(\Theta_{E}-\Theta_{L}\right)$$

where the coefficients in the equations govern the energy relaxation processes undergone by the three dynamical variables. In particular, *a* represents the electron gas heating rate and regulates the dynamics of the energy exchange from non-thermalised to thermal electrons. In the model, this is determined by comparison with first-principles calculations [178, 179] and estimated as an average over the accessible electronic energies from a rigorous expression of the e-e scattering rate ${\left(\tau_{ee}\right)}^{-1}$ [180], which scales quadratically with the pump photon energy. The coefficient *b* is instead the rate of excitation decay from the out-of-equilibrium nonthermal electronic population towards the metal phonons. Its expression directly stems from a rigorous definition of the e-ph relaxation time [178, 181] and is inversely proportional to the photon energy of the pump. In the equations for Θ_*E*_ [Eq. (8)] and Θ_*L*_ [Eq. (9)], the coefficients governing the variable dynamics are the electron gas and lattice heat capacities, *C*_*E*_ and *C*_*L*_ respectively, together with the thermal e-ph coupling coefficient, *G*. As long as ultrafast time scales are considered, *C*_*L*_ can be approximated as constant, while a dependence of the electronic heat capacity on Θ_*E*_ is required for a proper description of the behaviour of the thermalized carrier gas, which tends to increase its thermal inertia (i.e., *C*_*E*_ increases) as temperature increases. In typical excitation conditions, such dependence can be modelled as linear, i.e. *C*_*E*_ = *γ*_*E*_Θ_*E*_, by introducing a coefficient *γ*_*E*_ representing the linear carrier heat capacity constant [182]. However, in the case of high excitation levels (Θ_*E*_ > ∼3000 K), a super-linear dependence and more refined models should be considered [171, 172] to accurately estimate the dynamics of Θ_*E*_. The same applies to the e-ph coupling coefficient *G*, constant [183] for a relatively wide range of electronic temperatures (up to a few thousands of K), but increasing for even higher Θ_*E*_ [171]. As mentioned for the 2TM, these coefficients can be obtained from *ab initio* calculations, or retrieved phenomenologically by fitting experimental data.

Lastly, the driving term of the rate-equation model is *P*_abs_(*t*) [on the right hand side in Eq. (7)], representing the pump pulse power density (per unit volume) absorbed by the plasmonic structure. The analytical expression of this quantity is determined based on the pulse fluence and the metallic nanostructure absorption and inherits from the optical pulse the temporal profile (Gaussian-like, most generally) and duration. Note that the 3TM detailed in Eqs. (7)–(9) is suitable to investigate the ultrafast regime of photoexcitation up to several tens of ps. However, on longer time scales (from hundreds of picoseconds to nanoseconds), phonon–phonon scattering promotes the interaction between the metal lattice and the environment surrounding the nanostructure. Heat is released from the former to the latter, and a temperature increase within the environment is induced [62, 64]. This (much slower) photothermal dynamics, dominating the system optical response on the longer time scales, is the mechanism underlying the application of plasmonic nano-objects as efficient sources of heat. To account for this further energy flow channel, suitable extensions of the 3TM towards a four-temperature model formulation, which includes the temperature of the environment, should be considered [98, 184]. Moreover, upon the appropriate modifications of the equations, the conceptual approach behind the 3TM can be extended beyond plasmonics, and applied e.g. to semiconductor-based nanostructures. Akin to metals, light pulses photogenerate electron-hole pairs, which undergo ultrafast relaxation towards the lattice. The cascade of processes initiated by photoexcitation can then be accurately described by a 3TM, as it has been shown for heavily-doped semiconductor [185, 186] and even all-dielectric [187–189] nanostructures, as well as for epsilon-near-zero transparent conducting oxides [190, 191]. However, compared to plasmonic metals, the photophysics of semiconductors is more diverse, and various physical processes can take place, depending on e.g. the band structure and density of states of the considered material. As a result, in general, the precise formulation of the rate equations may change, according to the specific excitation/relaxation channels available.

Finally, we mention an additional approach, originally proposed in ref. [178] to include the contribution of non-thermal electrons in the 2TM, and referred to as the extended two-temperature model (E2TM). Conceptually, akin to the 3TM, the E2TM includes an alteration in the dynamics of the electron and phonon temperatures due to the presence of a non-thermalized fraction of the electronic population. Differently from the 3TM, this is done implicitly, i.e. without defining a further variable (*N*) and its corresponding rate equation. Rather, in the E2TM, the source terms for the only two dynamical degrees of freedom, i.e. Θ_*E*_ and Θ_*L*_, are expressed so as to account for an effective delayed energy flow due to non-thermal carriers. Specifically, they are written as time-convolution integrals between the exciting light pulse temporal shape and (e-e and e-ph) driving kernels derived from the BE. Compared to the 3TM, the formulation proposed in the E2TM allows for resolving the dependence on the electron energy of the e-e and e-ph scattering rates (washed out by the 3TM, assuming the same time constant for the relaxation processes of non-thermal electrons). This additional feature is achieved at the expense of a slightly more complex formalism in the model. We also note that, with the same rationale behind the E2TM, a more sophisticated dynamical multi-temperature model has been recently reported. It is referred to as the quantum 2TM, and refines the expression of the hot carrier generation rates based on quantum arguments [103, 114].

### 4.2 The spatio-temporal 3TM

Among the foundational assumptions of the 3TM, the most conventional formulation of the model considers a spatially homogeneous absorption of light across the nanostructure. In formulas, a uniform absorption amounts to express the dissipated power density, *P*_abs_ in Eq. (7), as function of time only. This in turn translates into disregarding the space-dependence of the three dynamical variables of the model. This hypothesis is justified as long as sufficiently small NPs are investigated. When the characteristic dimensions of the nano-object are much smaller compared to the optical wavelength, the QSA applies. According to theoretical predictions, the fields (hence the electromagnetic power density) within the NP can be considered as uniform. This assumption fails, however, when one investigates (i) larger nanostructures [192–194]; (ii) specific excitation conditions [195, 196] or (iii) complex shapes featuring e.g. tips, edges or sharp geometries [197–199]. In these conditions, neglecting the spatial effects of light–matter interaction across the NP becomes quantitatively and even qualitatively inadequate. A suitable modelling approach should start by introducing the spatial dependence of the optical excitation, beyond the QSA. This can be readily done by using standard computational tools, such as finite element method (FEM) or finite-difference time-domain (FDTD) simulations. From the spatial dependence of the electromagnetic fields, one can derive the precise pattern of light absorption within a nanostructure of arbitrary size and shape. To include this information in the 3TM, the expression of the absorbed power density *P*_abs_ can be recast to become a space- and time-dependent quantity, which models the photoexcitation accounting for the effects of e.g. electromagnetic hot spots, spatially localized field enhancements, the exact geometrical configuration of complex structures, or the specific pattern of the excited plasmonic modes. Specifically, expressing *P*_abs_ is straightforward by assuming that: (i) the excitation pulse duration is long enough to contain a large number of optical cycles (that is, by applying the slowly varying envelope approximation, which is generally fulfilled by optical pulses in the visible); and (ii) the pulse fluence is low enough to disregard nonlinear intra-pulse self-actions and to consider its absorption in the linear regime. As such, the pattern of *P*_abs_ is simply the one in the stationary regime and fixed in space, with its intensity modulated in time according to the pulse temporal profile. More sophisticated models have been proposed, pursuing a self-consistent (yet approximated) description of the nonlinear light–matter interaction [200, 201]. Results predict a quantitative mismatch with the linearized formulation, without distortion in the excitation dynamics (mostly because of the typically limited delay effects induced in plasmonic nanostructures), confirming the soundness of the linear approach.

Considering a space-dependence of the instantaneous absorbed power density entails introducing the spatial degrees of freedom in the model. Driven by a spatio-temporal excitation, the three energetic variables of the 3TM will indeed evolve in time and space, featuring transient spatial inhomogeneities at the deep sub-wavelength scale. To do so, partial differential equations should replace ordinary ones, and transport phenomena, involving electrons and phonons, have to be included. Following the 3TM thermodynamics-like treatment, Fourier-like diffusion terms represent the most straightforward choice. In this framework, the thermal conductivity of the metal, *κ*_*L*_, and that of the electronic population, *κ*_*E*_, regulate the phonon and thermalized carrier diffusive transport. Diffusion of non-thermal electrons has also been theoretically reported [202] and proposed to be governed by the same *κ*_*E*_ as for thermal electrons [203]. In the most general case, however, e-e scattering limits the lifetime of non-thermal carriers, which experience full relaxation on timescales comparable with the diffusion characteristic time. The latter process is therefore expected to be less impactful than the former on the dynamics of *N* and is therefore disregarded. The model, that we have referred to as the inhomogeneous three-temperature model (I3TM) in previous reports [58], reads as follows:

(10) $$\frac{\partial N\left(r,t\right)}{\partial t}=-aN-bN+P_{\text{abs}}\left(r,t\right)$$

(11) $$C_{E}\frac{\partial \Theta_{E}\left(r,t\right)}{\partial t}=-\nabla \cdot \left(-\kappa_{E}\nabla \Theta_{E}\right)-G\left(\Theta_{E}-\Theta_{L}\right)+aN$$

(12) $$C_{L}\frac{\partial \Theta_{L}\left(r,t\right)}{\partial t}=\kappa_{L}\nabla^{2}\Theta_{L}+G\left(\Theta_{E}-\Theta_{L}\right)+bN$$

where the explicit dependence on both time and space for *N*(**r**, *t*), Θ_*E*_(**r**, *t*) and Θ_*L*_(**r**, *t*) have been dropped on the right-hand side of the three equations for the sake of conciseness. To solve this set of spatio-temporally coupled partial differential equations, one can employ standard numerical methods based on time-stepping (e.g. Euler or Runge–Kutta) algorithms, provided the space and time steps in the solver are suitably chosen.

We conclude our introduction to the (I)3TM by highlighting the main limitations of such a modelling approach. Contrary to the 2TM, the (I)3TM includes the dynamical contribution of non-thermal carriers, without assuming instantaneous thermalization of photoexcited electrons. However, by disregarding the dependence of the e-e scattering rate on the electronic energies, it is not able to account for energy-resolved relaxation of hot carriers (see e.g. ref. [204] for a close comparison between the 3TM and the E2TM, the latter being the simplest variant of the 3TM able to include such effect). Additionally, by coupling directly the electromagnetic dissipated power density to the energetic degree of freedom inherent to non-thermal electrons, the model assumes instantaneous dephasing of the plasmon. The (electronic energy-dependent) mechanisms of hot carrier generation following the plasmon decay are therefore neglected, and no distinction between the different possible processes (either interband or intraband absorption, or via excitation of the plasmon) is made. Note also that, according to Eqs. (10)–(12), spatial diffusion of non-thermalized electrons is not accounted for. Finally, as a rate-equation model, the (I)3TM is intrinsically not suited to describe coherent effects of photoexcitation. Despite these limitations, such a modelling approach is indeed able to reproduce with remarkable accuracy the ultrafast dynamics observed experimentally, as well as to provide quantitative predictions of the optical behaviour of complex systems, as we will show in the following sections.

### 4.3 Optical nonlinearities for permittivity modulation

The 3TM (or, equivalently, its inhomogeneous extension) details the (spatio-)temporal evolution of the energetic degrees of freedom of the photoexcited plasmonic nanostructure, providing information on the dynamics of the hot carrier population. However, further steps in the model are required to determine the dynamics of the transient optical observables as, e.g., transmission or reflection, namely the outcomes of typical ultrafast spectroscopy measurements. First, one needs to establish how the internal variables determined with the 3TM affect the permittivity of the plasmonic material and contribute to the modulation of the optical response of the system. Indeed, both *N* and Θ_*E*_ drive modifications in the energy distribution of bound electrons, entailing a modulation of the metal interband permittivity, while Θ_*L*_ affects free electrons in the conduction band, changing thus the intraband term of *ɛ*.

More specifically, values of *N* and Θ_*E*_ in out-of-equilibrium conditions (*N* > 0, Θ_*E*_ > Θ_0_) dictate a variation in the electron energy distribution, Δ*f*(*E*, *t*), corresponding to a reduction (increase) of the occupation probability of the electronic states below (above) the Fermi energy, which in turn entails an increased (decreased) absorption for transitions involving final states below (above) *E*_*F*_. This mechanism translates into a variation of the imaginary part of the metal permittivity, Δ*ɛ*′′, accounting for the modified absorption for the interband transitions [205–207] in the metal (for instance, the 5*d*–6*sp* transitions in Au). For non-thermal carriers, the occupancy distribution modulation can be expressed as [94, 175]:

(13) $$\Delta f_{N}\left(E,t\right)=\delta_{N}\left(E\right)N\left(t\right),$$

where *δ*_*N*_(*E*) is a double-step-like function [175, 178], extending from −*hν* to +*hν* (*hν* being the excitation photon energy) around *E*_*F*_, broadened by temperature (and even further by broadband pump pulses [91]), and formally given by:

(14) $$\begin{array}{ll} \delta_{N}\left(E\right) & =\frac{1}{A}\left\{f\left(E-h\nu ,\Theta_{0}\right)\left[1-f\left(E,\Theta_{0}\right)\right]\right.\\ & \;\left.-f\left(E,\Theta_{0}\right)\left[1-f\left(E+h\nu ,\Theta_{0}\right)\right]\right\}, \end{array}$$

with *A* being a constant derived from the energy conservation law [178]. Note that expressing Δ*f*_*N*_ as the product in Eq. (13) implies that its spectral shape is constant in time and determined by the functional form of *δ*_*N*_(*E*), while its dynamics is precisely the same as *N*. Such a formulation corresponds to assuming that non-thermalized carriers undergo relaxation with the same time constant, regardless of their energy.

On the other hand, for thermalized electrons, the variation in the energy occupancy distribution can be expressed [175] as a difference between the excited and the equilibrium Fermi–Dirac distribution, namely:

(15) $$\Delta f_{\Theta_{E}}\left(E,t\right)=f\left[E,\Theta_{E}\left(t\right)\right]-f\left(E,\Theta_{0}\right).$$

The effect of an increased electronic temperature on the metal interband transitions is, as for non-thermal carriers, an increased (reduced) absorption for transitions towards final states below (above) the Fermi energy. This mechanism is commonly referred to as *Fermi smearing*, since the electronic occupation probability gets indeed smeared around *E*_*F*_ for increasing Θ_*E*_.

To translate variations of the electronic distribution into changes of the optical response of the plasmonic material, a semiclassical model of the thermo-modulational nonlinearities in metals [106, 107] presiding over the optical transitions in the solid state [205–208] can be implemented, relating Δ*f*_*N*_ and $\Delta f_{\Theta_{E}}$ to variations of the complex dielectric permittivity, Δ*ɛ*_*N*_ and $\Delta \varepsilon_{\Theta_{E}}$ respectively. In essence, a modified electron distribution corresponds to a change in the number of available direct transitions between valence and conduction bands. This mechanism can be accounted for by considering variations in the so-called joint density of states (JDOS), which quantifies the number of existing couples of states (per unit volume) within the first Brillouin zone (1BZ) with energy separation equal to the probe photon energy (and, as such, responsible for interband absorption). Formally, this quantity, which is a function of the probe wavelength *λ*, is given by [206]:

(16) $$\Delta J_{N\left(\Theta_{E}\right)}\left(\lambda ,t\right)=\overset{E_{\max}}{\underset{E_{\min}}{\int }}D\left(E,\lambda \right)\Delta f_{N\left(\Theta_{E}\right)}\left(E,t\right)dE,$$

where *D*(*E*, *λ*) is the energy distribution of the JDOS of the considered optical transition [207]. In the case of the prototypical plasmonic material Au, the dominant interband transition is the one near the *L* point in the 1BZ, followed by one at the *X* point, which may also be included in the model (here neglected in the following for clarity). For a focussed discussion of the details of the calculations, see e.g. ref. [107] and the references therein, providing numerical values for effective masses, energy gaps, dipole matrix elements and integration limits *E*_min_ and *E*_max_ for Au. Then, once $\Delta J_{N\left(\Theta_{E}\right)}$ is determined, the corresponding modification of the imaginary part of the interband permittivity can be expressed as:

(17) $$\Delta \varepsilon_{N\left(\Theta_{E}\right)}^{\prime \prime }\left(\lambda ,t\right)=\frac{e^{2}\lambda^{2}}{12\pi \varepsilon_{0}m^{2}c^{2}}|p_{L}{|}^{2}\Delta J_{N\left(\Theta_{E}\right)}\left(\lambda ,t\right),$$

with *m* and *e* the free electron mass and charge respectively, *ɛ*_0_ the vacuum permittivity and *c* the speed of light, while *p*_*L*_ is the electric-dipole matrix element of the considered (here around the *L* point only) transition. Kramers–Kronig relations allow then to straightforwardly retrieve the real part of the metal permittivity modulation $\Delta \varepsilon_{N\left(\Theta_{E}\right)}^{\prime }\left(\lambda ,t\right)$ accompanying the imaginary component as given in Eq. (17).

Different mechanisms instead preside over the changes in the intraband permittivity, which follow an increase in lattice temperature. First, the e-ph scattering rate increases with Θ_*L*_, translating into an increase in the Drude damping factor, Γ. Moreover, when heated up, the metal undergoes a thermal expansion, entailing a decrease in the free carrier density and subsequent decrease of the plasma frequency, *ω*_*p*_. Regarding the first effect, various modelling approaches are viable to express the variation of the Drude damping. A simple expression depending on the probe frequency was proposed in ref. [209], reading:

(18) $$\Delta \Gamma \left(t\right)=\beta {\left(\hbar \omega \right)}^{2}\Delta \Theta_{L}\left(t\right),$$

with *β* a constant coefficient characteristic of the considered metal [209] and *ω* = 2*πc*/*λ* the probe angular frequency. Alternative formulations were proposed e.g. in ref. [210] or in ref. [211] following Holstein’s model [212]. Note that, in the presence of high electronic temperatures, the e-e scattering rate may also change, its dependence on Θ_*E*_ having been derived in ref. [213]. Generally, for an intraband permittivity expressed as a Drude term, $\varepsilon_{D}\left(\omega \right)=1-\omega_{p}^{2}/\left(\omega^{2}+i\Gamma \omega \right)$ , the change corresponding to a variation of the damping factor reads:

(19) $$\Delta \varepsilon_{\Gamma }\left(\omega ,t\right)=\frac{i\omega_{p}\Delta \Gamma \left(t\right)}{\omega \left(\omega +i\Gamma \right)\left[\omega +i\Gamma +i\Delta \Gamma \left(t\right)\right]}.$$

On the other hand, the variation of plasma frequency due to an increase in lattice temperature can be determined by recalling that the plasma frequency scales with the square root of the free electron concentration. By thus referring to the volume thermal expansion coefficient *α*_*V*_ [93, 182], the plasma frequency variation reads [214, 215]:

(20) $$\Delta \omega_{p}\left(t\right)=-\frac{1}{2}\alpha_{V}\omega_{p}\Delta \Theta_{L}\left(t\right),$$

from which the corresponding permittivity variation can be written as:

(21) $$\Delta \varepsilon_{\omega_{p}}\left(\omega ,t\right)=-\frac{\Delta \omega_{p}\left(t\right)\left[\Delta \omega_{p}\left(t\right)+2\omega_{p}\right]}{\omega \left(\omega +i\Gamma \right)}.$$

In the most general case, both ΔΓ and Δ*ω*_*p*_ affect the intraband permittivity. $\Delta \varepsilon_{\Theta_{L}}$ therefore comes from a combination of the two effects driven by an increase of the lattice temperature and is expressed as the sum of Δ*ɛ*_Γ_ from Eq. (19) and $\Delta \varepsilon_{\omega_{p}}$ from Eq. (21).

To eventually determine the total photoinduced modulation of the metal permittivity, all the terms computed above are summed up, giving a complex-valued $\Delta \varepsilon \left(\lambda ,t\right)=\Delta \varepsilon_{N}+\Delta \varepsilon_{\Theta_{E}}+\Delta \varepsilon_{\Theta_{L}}$ . Note that each of these three contributions has a characteristic wavelength dependence and evolves in time with a dynamics inherited from the corresponding internal degree of freedom driving the modulation (although $\Delta \varepsilon_{\Theta_{E}}$ can possibly exhibit a nontrivial dynamics when the system is excited beyond the perturbative regime [216]). A complex interplay of effects results therefore from their superposition, with relative weights varying in time and across the probe spectrum [99, 204, 217].

### 4.4 Calculation of the spectroscopic observables

As detailed in Section 3, TA spectroscopy represents a very powerful experimental technique for studying the ultrafast dynamics of hot carriers in photoexcited plasmonic nanostructures. Modelling TA spectroscopy measurements is thus of key relevance since it allows both (i) a refined interpretation of experimental results; and (ii) a clear-cut validation of the (I)3TM. The differential transmission signal, Δ*T*/*T*, can be directly determined by iterating the calculation of the transmission of the nanostructure time by time over the spectral range of interest, considering the modified optical properties of the plasmonic nanomaterial according to a permittivity *ɛ*(*λ*, *t*) = *ɛ*_0_(*λ*) + Δ*ɛ*(*λ*, *t*), where *ɛ*_0_(*λ*) is the unperturbed permittivity. This can be done via full-wave FEM-based numerical simulations, as well as via analytical expressions based on the QSA. However, in the weak photoexcitation regime, it is possible to consider the photoinduced variations of permittivity to be much smaller than the static permittivity of the active material. In this framework, a perturbative approach can be developed, expressing the differential transmission as:

(22) $$\frac{\Delta T}{T}\left(\lambda ,t\right)=c^{\prime }\left(\lambda \right)\Delta \varepsilon^{\prime }\left(\lambda ,t\right)+c^{\prime \prime }\left(\lambda \right)\Delta \varepsilon^{\prime \prime }\left(\lambda ,t\right),$$

where *c*′(*λ*) and *c*′′(*λ*) are suitable spectral coefficients for transmission, approximating the first order partial derivatives of *T* with respect to variations of permittivity, normalized to the static transmission *T*(*λ*). In formulas, these coefficients can be computed as:

(23) $$c^{\prime }\left(\lambda \right)=\frac{1}{T\left(\lambda \right)}\frac{\bar{T}\left(\lambda \right)-T\left(\lambda \right)}{\bar{\Delta \varepsilon^{\prime }}}$$

and similarly for *c*′′ (by replacing $\bar{\Delta \varepsilon^{\prime }}$ by $\bar{\Delta \varepsilon^{\prime \prime }}$ ). In the formula above, $\bar{\Delta \varepsilon^{\prime }}$ (or $\bar{\Delta \varepsilon^{\prime \prime }}$ ) is a constant representing a small fictive modification of the material permittivity over the investigated spectrum, producing a perturbed transmission $\bar{T}\left(\lambda \right)$ . It can be set to ∼1 in metals in order to mimic typical low-perturbation-regime changes of permittivity. Note that, upon the appropriate definition of the spectral coefficients, the same perturbative approach can be applied to different optical quantities, e.g. reflection, or the extinction cross-section.

**Figure 4:**
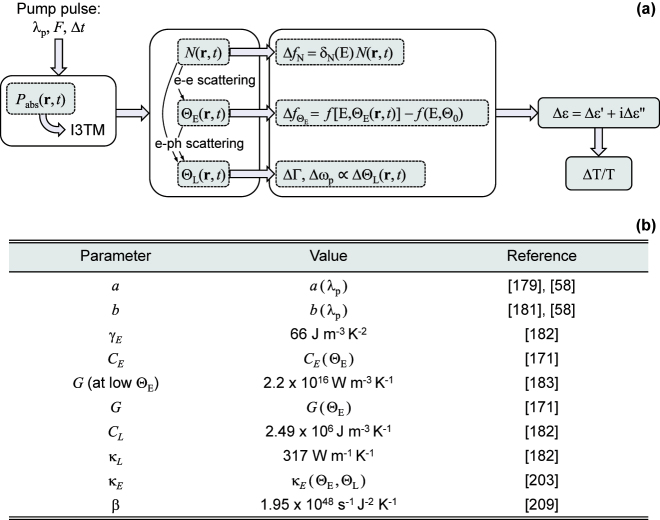
Modelling photoinduced optical nonlinearities driven by hot carrier dynamics. (a) Schematic of the implementation algorithm underlying our modelling approach, which provides the dynamics of typical optical quantities (e.g. the differential transmittance Δ*T*/*T*) retrieved by ultrafast TA spectroscopy, by describing the internal relaxation processes undergone by hot carriers with the I3TM and corresponding nonlinear modulation of the metal permittivity starting from the pump pulse wavelength *λ*_*p*_, fluence *F* and temporal duration Δ*t*. (b) Table of the typical values and references for the most relevant parameters employed in the I3TM when applied to Au.

To conclude this section dedicated to the (I)3TM-based description of hot carrier dynamics and corresponding optical nonlinearities, we visually summarize our full modelling approach in Figure 4a. Additionally, Figure 4b displays a table collecting typical values and references for the most relevant parameters used in the model when applied to Au (among the most representative plasmonic materials). Importantly, the implementation of this algorithm allows us to straightforwardly examine the dynamics of the three energetic degrees of freedom separately, and thus to gauge unambiguously their relative contributions to the total optical modulation. This provides us with a powerful tool to unfold the measured signal, disentangling it in terms of the different modulation mechanisms following photoexcitation, as exemplified in the following section.

## 5 Representative results

### 5.1 Electron–electron scattering in thin gold films

Accessing the very early relaxation dynamics regulated by e-e scattering calls for a very high temporal resolution down to tens of femtoseconds. Here we exploit ultrafast TA spectroscopy to study the nonlinear response of a thin gold film, which represents the simplest example of a nanostructured metal. In our experiment, a 30-nm-thick gold film deposited on a fused silica substrate is excited by ultrashort pulses generated by a NOPA, with 10–12 fs duration and peak photon energy tunable in the 1.0–1.9 eV range (∼650–1240 nm). The differential transmission Δ*T*/*T* is probed by broadband ∼10-fs pulses, generated by a second NOPA, spanning 2.1–2.55 eV photon energies (i.e., 490–580 nm), thus covering the frequency range of gold interband transitions. Details on the experimental setup used can be found elsewhere [218–220].

**Figure 5:**
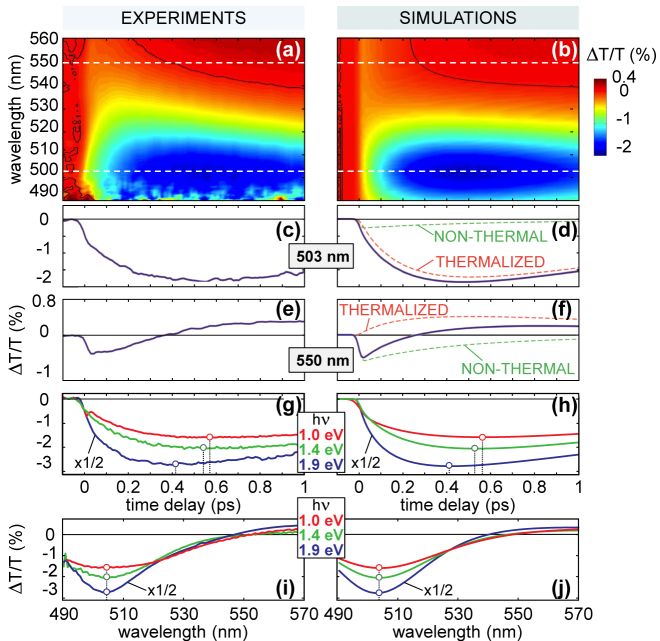
Ultrafast dynamics of hot electrons in Au thin films. (a–f) Δ*T*/*T* maps (a, b) and dynamics (c–f) at selected probe wavelengths (503 nm in c, d; 550 nm in e, f), obtained from experimental measurements (left panels) and numerical simulations (right panels) for a gold film pumped by a 12-fs pulse at 1.4 eV (∼885 nm), with incident pump fluence 430 μJ/cm^2^. Dashed lines in panels (d) and (f) represent the contributions to the total Δ*T*/*T* signal due to non-thermal (green) and thermalized (red) electrons, respectively. (g–j) Effects of pump photon energy. Experimental (g) and simulated (h) Δ*T*/*T* dynamics at 503 nm for different pump photon energies (corresponding to ∼1240 nm, 885 nm, 652 nm), together with experimental (i) and simulated (j) Δ*T*/*T* spectra at the time delay of the signal peak, i.e. 580, 540 and 410 fs for *hν* = 1.0, 1.4 and 1.9 eV, respectively. Adapted with permission from ref. [91]. Copyright 2012, American Physical Society.

Figure 5 shows the main results of our study [91]. The experimental Δ*T*/*T* map recorded for 1.4 eV (∼885 nm) pump photon energy is reported in Figure 5a. Right after the photoexcitation, a rather weak negative Δ*T*/*T* signal is observed at all probe photon energies, with rather flat frequency dependence, which is consistent with the ultrafast build-up of a broad non-thermal carrier distribution. Within 200 fs, a negative Δ*T*/*T* band grows and narrows considerably, peaking at ∼503 nm (2.465 eV), close to the onset of the interband transitions in Au (∼2.3 eV) [207]. Simultaneously, the Δ*T*/*T* signal turns positive in the red wing of the transient spectrum (*λ* > 540 nm, *hν* < 2.3 eV). The isosbestic line clearly shifts to shorter wavelengths with time (see black lines in Figure 5a). This complex spectral evolution reflects the interplay of e-e and e-ph scattering processes throughout electron thermalization, which can be followed in real time thanks to the combination of high temporal resolution and broad spectral coverage of our TA setup. The experimental data are modelled by the E2TM mentioned in Section 4.1 [178], which implicitly considers a population of non-thermal electrons induced by pump absorption and mediating the excitation of thermalized electrons and the metal lattice. Solving the E2TM, one can calculate the photoinduced change in the permittivity of gold, Δ*ɛ*(*λ*, *t*), as detailed in Section 4.3, and the resulting transmission change of the thin gold film using standard thin film formulas [221].

The results of the model are reported as Δ*T*/*T* map in Figure 5b and show a good quantitative agreement with the experimental data without any free parameter. A direct comparison between measured and simulated signals at selected wavelengths (Figure 5c and d at 503 nm, Figure 5e and f at 550 nm, respectively) is also shown, including a disentanglement of the contributions to the Δ*T*/*T* dynamics arising from non-thermal (green curves) and thermalized (red curves) carriers. In particular, we find that the sign change of the Δ*T*/*T* signal observed around 550 nm is due to the interplay between the contributions of non-thermal electrons, which dominate at early times (see the green line in Figure 5f), and thermal electrons, which dominate at later times (see the red line in Figure 5f).

We also investigate the effects of pump photon energy on the e-e scattering dynamics, which are summarized in Figure 5g–j. We find that the Δ*T*/*T* maps recorded for 1 and 1.9 eV pump photon energies (i.e., ∼1240 nm and 652 nm, respectively) exhibit the same qualitative features as the one measured for 1.4 eV (i.e., 885 nm). However, the peak of the negative Δ*T*/*T* band, centred at ∼503 nm almost irrespective of the pump photon energy, is reached at different times. These delays get progressively shorter for increasing pump photon energy, going from ∼580 fs at 1 eV (1240 nm) to ∼410 fs at 1.9 eV (652 nm), as shown in Figure 5g. This variation of the thermalization dynamics with pump photon energy is also reflected in the different spectral widths of the peak at 503 nm probe wavelength (Figure 5i) and is accurately reproduced by our numerical model (Figure 5h and j), which also provides an explanation for the signal behaviour. The acceleration of the peak formation can indeed be related to the e–e scattering rate which governs the decay (rise) of the non-thermal (thermal) electrons. When the pump photon energy is higher, the temperature increase rate scales quadratically with *hν*, confirming the observed trend, and consistently with the fact that electrons with higher excitation energy above the Fermi level possess a faster scattering rate, as predicted by the Fermi liquid theory.

### 5.2 Plasmonic resonance dynamics in single gold nanoantennas

**Figure 6:**
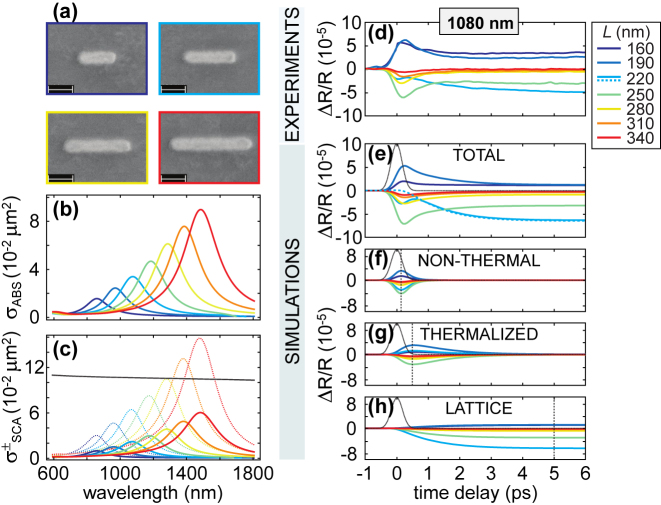
Ultrafast dynamics of hot electrons in Au single nanoantennas. (a) Scanning Electron Microscopy (SEM) images of the single Au nanoantennas of comparable width (50 nm) and different lengths *L*: 160 nm (dark blue), 220 nm (light blue), 280 nm (yellow) and 340 nm (red). Scale bar: 100 nm. (b and c) Simulated static spectra of the absorption (b) and both backward (solid lines) and forward (dotted lines) scattering cross-sections (c) of nanoantennas with different lengths. Black solid line represents the equivalent backward scattering cross-section from the substrate for a probe beam with 1-μm spot radius. (d–h), Experimental (d) and simulated (e) Δ*R*/*R* time traces at 1080 nm probe wavelength for nanoantennas of different length. The total simulated Δ*R*/*R* signal from each nanoantenna is further disentangled in terms of the contributions arising from non-thermal electrons (f) thermalized electrons (g) and the lattice (h). Dashed line in (e) represents the simulated Δ*R*/*R* for the 220-nm-long nanoantenna neglecting the contribution from non-thermal electrons. Dotted lines in (e–h) are the pump-probe cross-correlation assumed in the simulations (corresponding to the dynamics of the driving term in the 3TM). Vertical dashed lines in (f–h) indicate the time delay at which the disentangled Δ*R*/*R* signal is maximum. Adapted with permission from ref. [94]. Copyright 2015, American Chemical Society.

Among plasmonic nanostructures, gold nanorods (NRs) have been the subject of intensive investigation, in view of their peculiar optical response, strongly polarization-dependent and easily tunable by acting on their aspect ratio (see, e.g., ref. [65]). Moreover, the high scattering efficiency that NRs feature when excited at their longitudinal LSPR (electric field aligned to their long axis) makes them the prototype of a dipolar nanoantenna. Here we report the transient response of a series of isolated gold NRs fabricated by electron beam lithography, with the longitudinal LSPR tuned according to the length of the NR. Such study of the ultrafast behaviour of single nanoantennas requires the combination of TA spectroscopy with microscopy (see Section 3.2). Figure 6 shows our main results, obtained with NRs of 50 nm width and variable length *L* ranging from 160 nm to 340 nm (see scanning electron microscope images of some of the NRs in Figure 6a) [94]. Specifically, Figure 6b and c show the simulated absorption and (backward and forward) scattering cross-sections of each NR, respectively. The obtained static spectra clearly exhibit a red-shift of the resonant peak with increasing *L*, ranging from the visible to the near infrared. To investigate the dynamical behaviour of the NRs, each of them is pumped off-resonance at 780 nm with ∼100-fs pulses and a pump fluence of 63 μJ/cm^2^, and the differential reflection Δ*R*/*R* = *R*_ON_/*R*_OFF_ − 1 is recorded as a function of the pump-probe delay time, with *R*_ON_ (*R*_OFF_) the probe signal reflected by the excited (unperturbed) structure. Note that working in reflection in the pump-probe measurements introduces more challenges compared to transmission (also analysed, not shown here), because of e.g. the background noise from the substrate. The probe wavelength, tunable in the 850–1000 nm range and independently from the pump wavelength, is set to 1080 nm, corresponding to the spectral position of the resonance featured by one specific NR (the one with length *L* = 220 nm). As a result, varying the NR length corresponds to changing the plasmon-probe detuning, i.e. the spectral distance between the probe wavelength and the plasmonic resonance of the individual nanoantennas.

Figure 6d reports a series of Δ*R*/*R* dynamics for isolated NRs with different *L*. The experimental traces feature both positive and negative signs, depending on the NR longitudinal size. All the dynamics display a resolution-limited rise time of ∼300 fs, after which a monotonic decrease (in modulus) is observed, until the establishment of a long-living plateau, after ∼5 ps. The only exception is the NR with *L* = 220 nm (cyan traces in Figure 6), namely the one resonant with the probe (i.e. with zero detuning). The dynamics of this NR exhibits indeed a non-monotonic behaviour, with a dip at ∼0.1 ps, followed by a peak at ∼0.4 ps before the monotonic decrease towards the long-living plateau, giving a higher signal compared to the other NRs. To rationalize the complex spectro-temporal features observed in the experiments and explain the behaviour of each nanoantenna, we adopt the 3TM-based description of the phenomena involved in the nanoantenna photoexcitation (cfr. Sections 4.1 and 4.3) integrated with FEM simulations of the electromagnetic response of isolated NRs. The transient extinction cross-section spectra of the nanoantennas are modelled according to a variant of the perturbative approach detailed in Section 4.4. Figure 6e reports the results of the numerical model of the Δ*R*/*R* signal of the single NRs with varying length.

Comparing experimental (Figure 6d) and simulated (Figure 6e) traces suggests that the model not only captures the main qualitative features of the Δ*R*/*R* collected from the different NRs, but it also displays a nearly quantitative match. The agreement between measured and simulated traces is achieved for both the onset of the signal peaks/dips, as well as the long-lived plateaus, confirming that our model is suited to describe the observed dynamics on both the timescales of e-e (sub-ps) and e-ph (a few ps) scattering. The measured signal is precisely reproduced even in its less trivial dynamics, as the one of the NR with *L* = 220 nm (cyan curves in Figure 6d–h). Furthermore, our accurate model provides a deeper interpretation of the measured differential reflection, by disentangling the dynamics of the total Δ*R*/*R* signal (Figure 6e) into the contributions arising from non-thermal electrons (Figure 6f), thermalized carriers (Figure 6g), and the metal lattice (Figure 6h). Such an analysis allows us to unambiguously assign the observed features to distinct photophysical processes. The lattice contribution (Figure 6h), driven by the temporal evolution of the Au phonon temperature increase, is shown to be the predominant after ∼1.5 ps. On the other hand, within the first ps, the most relevant contributions arise from the hot, both non-thermal (Figure 6f) and thermalized (Figure 6g), carriers, with the former following a much faster dynamics (estimated relaxation time ∼260 fs) than the latter. Moreover, the two distinct permittivity modulation terms arising from the photoexcited electronic population contribute differently to the transient Δ*R*/*R* at the probe wavelength, due to the differences in their probe wavelength dependence. In particular, when the plasmon-probe detuning is close to zero (i.e. for the NR with *L* = 220 nm), this effect becomes the most relevant. Non-thermal and thermalized carriers indeed contribute to the total signal with opposite signs (compare cyan curves in Figure 6f and g), and their interplay is responsible for the dip-peak oscillation observed in the dynamics for this specific nanoantenna within the first hundreds of femtoseconds. This feature represents the fingerprint of the specific contribution of non-thermal electrons, while it is washed out when considering thermalized carriers only (see dotted line in Figure 6e).

### 5.3 Transient optical symmetry breaking in plasmonic metasurfaces

We have previously discussed the effect of hot electrons on the transient permittivity of plasmonic nanostructures, which can be exploited to modulate their optical response on an ultrafast timescale. However, by a rational design, the nanoscale spatial inhomogeneities of hot carriers can have a pivotal role in the nonlinear response of metallic nano-objects. Here we introduce two nanostructures in which the spatio-temporal hot electron dynamics drives a transient symmetry breaking enabling nanophotonic functionalities on the ultrafast timescale.

**Figure 7:**
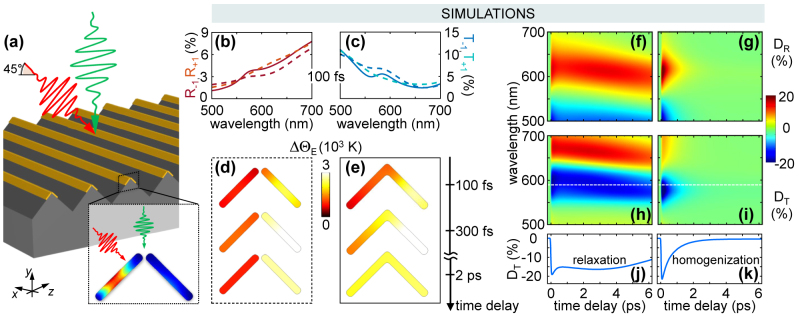
Design of a plasmonic metagrating for ultrafast diffraction management. (a) Sketch of the plasmonic metagrating, consisting of a 1D array (with a periodicity of 800 nm) of cross-polarized Au nanostrip meta-atom dimers (each strip being 165 nm in length and 22.5 nm in width). A linearly-polarized pump pulse at 600 nm impinging at 45° (red) and a broad probe pulse at normal incidence (green) are considered. The inset displays the absorption pattern at 50 fs time delay across the meta-atom. (b and c) First-order transient reflection *R*_±1_ (b) and transmission *T*_±1_ (c) spectra at 100 fs time delay (dashed lines), compared with static (degenerate) spectra (solid lines). (d and e) Spatial distribution of the electronic temperature increase in the disconnected dimer nanostrip (d) and the connected variant (e) at selected time delays. (f–k) Maps of the *D*_*R*_ (f, g) and *D*_*T*_ (h, i) figures of merit, together with the *D*_*T*_ dynamics at 590 nm (j, k) for the disconnected (f, h, j) and connected (g, i, k) nanostrip meta-atom configurations. Adapted with permission from ref. [222]. Copyright 2020 Author(s), licensed under a CC-BY Creative Commons Attribution 4.0 License.

In a first study, we present the design of a plasmonic metagrating made of symmetric meta-atoms to achieve ultrafast control of the nanostructure diffraction orders [222]. Our results are summarized in Figure 7.

We consider a metagrating consisting of a 1D array of Au cross-polarized nanostrip dimers, supported by a dielectric substrate (Figure 7a). The metagrating response under p-polarized plane wave excitation at normal incidence (electric field along the *z*-axis) features a resonant absorption at ∼580 nm, and non-zero +1 and −1 diffraction orders, both in reflection and transmission (shown as solid lines in Figure 7b and c, respectively). In unperturbed conditions, the two ±1 reflection (transmission) orders are degenerate at normal incidence, because of the left-right optical symmetry of the meta-atom. To break this symmetry on an ultrafast timescale, we simulate a pump-probe experiment, where an ultrashort p-polarized pump pulse impinges with an angle of 45°. In these conditions, photoexcitation produces a non-uniform pattern of the near fields across the dimer meta-atom, as one arm is directly exposed to the incoming light, while the other is shaded from radiation. The resulting spatial distribution of the absorbed electromagnetic power density within each plasmonic nano-unit, shown in the inset of Figure 7a, is highly inhomogeneous. Photoinduced hot carriers are thus generated with a pattern reminiscent of that of absorption, and then diffuse over time and space, driving a space-dependent transient modulation of the material permittivity. We use the I3TM introduced in Section 4.2 to describe the photoinduced processes following pump absorption. Then, when a p-polarized probe pulse at normal incidence interrogates the excited nanostructure, it experiences a metagrating made of optically asymmetric unit cells. The photoinduced symmetry breaking lifts the degeneracy between the +1 and −1 diffraction orders, with the dynamical *R*_+1_ and *R*_−1_ reflection spectra (dashed lines in Figure 7b, for a pump-probe delay of 100 fs) deviating from the degenerate $R_{\pm 1}^{0}\left(\lambda \right)$ spectrum (solid lines in Figure 7b). The same transient imbalance of the ±1 orders occurs in transmission, as detailed in Figure 7c. Note that the predicted effect is fully reversible, and the recovery of a symmetric state is conditioned by the equilibration of electronic temperatures between left and right nanostrips. For the considered geometry, this occurs within few tens of picoseconds since a long-lasting asymmetry remains, after the ultrafast homogenization of ΔΘ_*E*_ within each strip separately (see Figure 7d). The e-ph relaxation is required to retrieve the optical symmetry. To overcome this bottleneck and speed up the recovery, we design a variation of the meta-atom where the two arms are connected. Figure 7e shows the impact of such a slight geometrical change, enabling complete homogenization of ΔΘ_*E*_ in ∼2 ps, as energy readily flows from the more excited arm strip to the one less exposed to the pump pulse well before e-ph relaxation. Figure 7f and g (7h and i) compares the results of our dynamical simulations for the connected and disconnected configurations in terms of *D*_*R*_ (*D*_*T*_), a metric quantifying the ±1 diffraction order imbalance in reflection (transmission) defined as the normalized difference $D_{R}=\left[R_{+1}\left(\lambda ,t\right)-R_{-1}\left(\lambda ,t\right)\right]/2R_{\pm 1}^{0}\left(\lambda \right)$ (and similarly for *D*_*T*_, expressed in terms of ±1 transmission orders). Time traces of *D*_*T*_ (at the wavelength of the signal peak) are also shown in Figure 7j and k, illustrating the substantial speed-up of the recovery when hot carrier dynamics is dictated by their homogenization (Figure 7g, i, k) instead of their relaxation (Figure 7f, h, j).

A second study of transient optical symmetry breaking caused by the spatial transients of hot carriers is presented in the following, demonstrating a novel strategy to control the polarization state of light with unprecedented speed. Our nanostructure design and theoretical modelling are here combined with the experimental verification of the predicted transient photoinduced anisotropy, paving the way to ultrafast dichroic devices for all-optical modulation and reconfiguration of light [58, 223].

**Figure 8:**
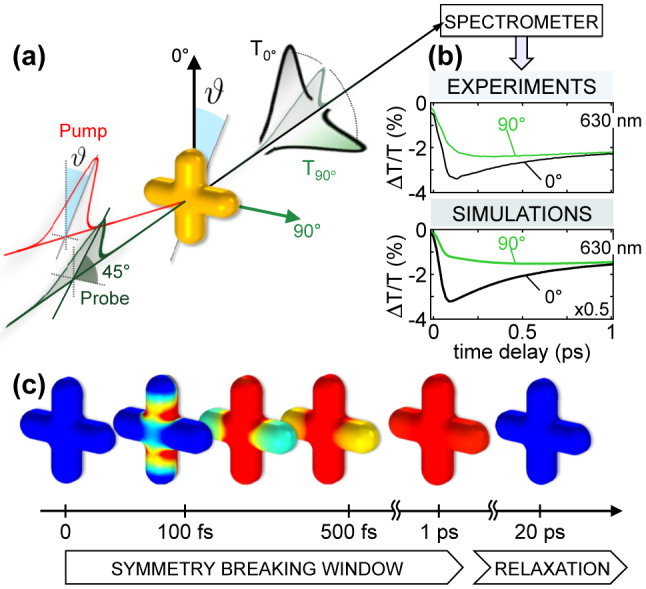
Design of a plasmonic metasurface for ultrafast symmetry breaking. (a) Sketch of our design concept, using a linearly-polarized pump pulse with fixed polarization angle *ϑ*, and probe pulses with linear polarization at 45° to the nanocross arm, analyzed along directions at 0° (black) and 90° (green). Adapted with permission from ref. [223]. Copyright 2022 Author(s), licensed under a CC-BY Creative Commons Attribution 4.0 License. (b) Experimental (top) and simulated (bottom) Δ*T*/*T* signal at 630 nm collected along the 0° (black) and 90° (green) direction of detection (for a pump polarization angle *ϑ* = 0°). (c) Sketch of the transient permittivity pattern (|Δ*ɛ*′′(**r**, *t*)|, evaluated at around 630 nm) evolving over time at the nanoscale (for a pump polarization angle *ϑ* = 0 ). Adapted with permission from ref. [58]. Copyright 2020, Springer Nature.

The rationale behind this concept is illustrated in Figure 8. We consider a plasmonic symmetric nanocross with arms of equal length. Due to the nanostructure C_4_-symmetry, its optical response is isotropic when illuminated with linearly polarized light at normal incidence, i.e. invariant under rotation of the angle *ϑ*, formed between the light polarization direction and the cross vertical arm (see Figure 8a). Conversely, a significant dependence on the polarization angle is observed for the near fields across the nanoparticle and, therefore, for its light absorption pattern, characterized by a well-structured spatial distribution which changes with *ϑ*. By reducing the nanocross to a superposition of two orthogonal and frequency-degenerate nanorods, we interpret the polarization-dependent absorption patterns as a combination of two plasmonic modes reminiscent of the longitudinal resonance of each single arm. When *ϑ* is either 0° or 90°, light mostly excites either the vertical or the horizontal arm, while in-between angles induce an imbalanced spatial configuration with intermediate symmetry. In all conditions (except for *ϑ* = 45°, when contributions of the two arms have equivalent weights and achieve a critical, perfect balance), the C_4_-symmetry of the nanocross is broken.

Hot carriers are photogenerated within the nanocross with a non-uniform spatial distribution dictated by *ϑ*, which translates into an anisotropic modification of the material permittivity on the ultrafast timescale. This makes the symmetry breaking of the electronic spatial configuration readily accessible by optical means. The fingerprint of the local permittivity modulation driven by hot carriers is a transient *ϑ*-dependent anisotropy, which can be revealed in a polarization-resolved TA spectroscopy experiment. In view of further developments of our approach towards real-world applications, we conceived an extended configuration of nanostructures, i.e. a metasurface consisting of a squared array, where the plasmonic nanocross is the nano-unit (meta-atom) repeating periodically in the plane. The resulting quasi-2D metamaterial exhibits optical properties inherited from the individual nanostructures, including the optical isotropy in unperturbed conditions, and a resonant behaviour regulated by the geometrical parameters of the single meta-atom.

In the experimental set-up used, the resonant absorption of the pump photogenerates hot carriers across the nanostructure. By tuning the direction *ϑ* of the pulse polarization, we control the symmetry of the spatial absorption pattern and the features of the transient nonlinear optical response. A delayed probe pulse, impinging at normal incidence with linear polarization at 45° to the nanocross arms, interrogates the optically excited structure over a broad range of visible wavelengths. The measured Δ*T*/*T* signal, as a function of the probe wavelength and the pump-probe time delay, is analysed along the polarization directions corresponding to the vertical (0°) and horizontal (90°) arms of the nanocross. This provides two distinct differential transmission signals, Δ*T*_0°_(*λ*, *τ*)/*T*(*λ*) = Δ_0_(*λ*, *τ*) and Δ*T*_90°_(*λ*, *τ*)/*T*(*λ*) = Δ_90_(*λ*, *τ*), with *T*(*λ*) the static one (insensitive to *ϑ*). An ultrafast anisotropy should manifest itself as a difference between Δ_90_ and Δ_0_ (see Figure 8b), hence we refer to the quantity Δ_90_ − Δ_0_ as a measure of the transient symmetry breaking. Since the effect is driven by the spatial inhomogeneities of hot carriers, its lifetime is dictated by the electron homogenization processes at the nanoscale. As illustrated in Figure 8c (for *ϑ* = 0°), in a few hundred femtoseconds the spatial diffusion of hot carriers restores a uniform distribution within the nanocross, which thus retrieves an excited yet isotropic state. This closes the temporal window for the photoinduced symmetry breaking and enables recovery of the initial isotropic configuration in less than 1 ps, well before complete relaxation of the system (tens of ps). As such, hot carrier spatio-temporal transients allow overcoming the speed bottlenecks of optical modulation caused by slower (e-ph and ph-ph) relaxation processes. Furthermore, the optically induced transient anisotropy can be controlled in intensity by tuning the polarization direction of the pump pulse. Active control and all-optical reconfiguration (up to a sign reversal) of the ultrafast response are achieved, with no need to change the geometrical design.

**Figure 9:**
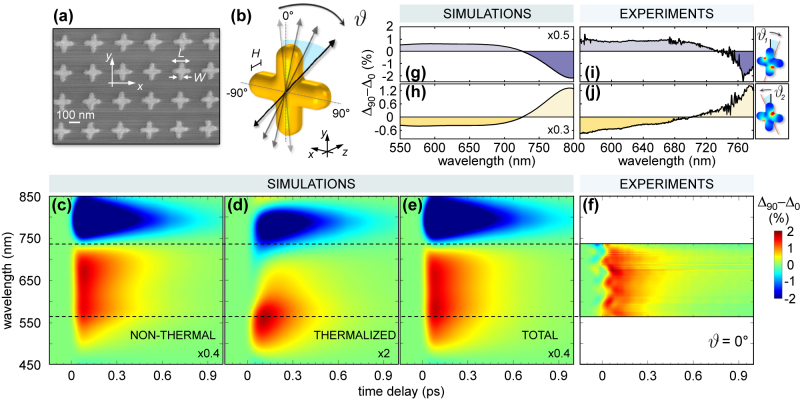
Ultrafast symmetry breaking for all-optical modulation and reconfiguration of light polarization. (a) SEM image of the metasurface. (b) Sketch of the light–matter interaction between the cross-shaped meta-atom and the linearly polarized light with varying polarization angle *ϑ*. (c–f) Simulated ultrafast photoinduced dichroism spectrum for pump polarization angle *ϑ* = 0°, disentangled in terms of the contributions arising from non-thermal carriers (c) thermalized carriers (d) together with the total effect (e) and the experimental signal (f) in the spectral range accessible by the measurement (fluence ∼400 μJ/cm^2^). Adapted with permission from ref. [58]. Copyright 2020, Springer Nature. (g–j) Simulated (g, h) and experimental (i, j) spectra of the dichroism evaluated at 100 fs time delay for a pump polarization angle of *ϑ*_1_ (g, i) and *ϑ*_2_ = *ϑ*_1_ + 90°. Adapted with permission from ref. [223]. Copyright 2022 Author(s), licensed under a CC-BY Creative Commons Attributions 4.0 License.

For an accurate estimation of the effect, we first develop a quantitative numerical model, combining full-wave FEM-based simulations, the I3TM, and the semiclassical modelling of the material optical nonlinearities (see Section 4 for details). With the design guidelines, we fabricated a plasmonic metasurface made of closely packed (270 nm periodicity) gold nanocrosses with arm length, width and height equal to *L* = 165 nm, *W* = 60 nm and *H* = 45 nm, respectively (see Figure 9a, showing a SEM image of the measured sample). To finally demonstrate the predicted effect, we set up the experimental apparatus to perform high-time-resolution (sub-10-fs), broadband and polarization-resolved TA spectroscopy measurements. According to the sketch in Figure 9b, the pump pulse polarization is rotated so to form a given angle *ϑ* with the nanocross vertical arm.

A first set of simulations and measurements is performed at *ϑ* = 0°, namely the optimal and most robust condition to photoinduce the symmetry breaking. The model reveals that on the 100-fs timescale, energy is released via e-e scattering from non-thermal carriers (mostly localized along the vertical arm) to the thermalized electrons, which inherit the spatial inhomogeneity and then undergo spatial diffusion over a timescale of a few hundreds femtoseconds. We note that e-ph and ph-ph scattering processes, taking place on a much longer ps timescale, are not relevant for the photoinduced anisotropy.

We compare the theoretical predictions with our polarization-resolved ultrafast TA experiments. The measured transient linear dichroism Δ_90_ − Δ_0_ is shown in Figure 9f and matches very well the simulations of Figure 9e over a broad probe spectral range (565–735 nm). Furthermore, the model enables to elucidate the main contribution to the anisotropy, arising mainly from non-thermal carriers (Figure 9c). Remarkably, thermalized electrons (Figure 9d) also contribute (to a lesser degree) to the transient dichroism, although the relaxation of the electronic temperature requires a timescale much longer than the symmetry-breaking window. The observed transient linear dichroism of ∼2% , comparable with the amplitude of the Δ*T*/*T* signals, is promising in view of applications to an ultrafast polarization modulator.

Finally, we show that our approach offers the further possibility to reconfigure the metasurface nonlinear response, thanks to the all-optical reshaping of the spatio-temporal distribution of hot carriers. We demonstrate the ability to tailor the intensity of the photoinduced anisotropy by measuring a sign reversal of the ultrafast dichroism by tuning the pump polarization direction. For instance, using a pair of pump pulses with polarizations angles, *ϑ*_1_ and *ϑ*_2_, differing by 90° results in opposite symmetries of the transient anisotropy, due to the polarization-sensitive spatial pattern of light absorption. In these two excitation conditions, the ultrafast dichroism shows comparable magnitude, but it experiences a switch in sign over the entire spectrum. Figures 9g–j illustrate this effect by reporting selected (simulated and measured) spectral cuts of the Δ_90_ − Δ_0_ signal at a pump-probe delay of 100 fs. Spectra clearly show that the positive/negative bands switch in sign when moving from *ϑ*_1_ (Figure 9g and i) to *ϑ*_2_ (Figure 9h and j), demonstrating the all-optical reconfiguration of the nanostructure response up to a full sign reversal by simple rotation of *ϑ*.

## 6 Conclusions and outlook

In this review we have briefly introduced the fundamental concepts of plasmonics and hot carrier physics, by discussing respectively the origin of the resonant behaviour of metallic nanostructures (in the simplest case of a small nanosphere), and the electronic relaxation processes taking place in photoexcited metals at the nanoscale. We then presented two among the most powerful experimental techniques to study the dynamics of photoexcited electrons in plasmonic nanostructures, namely ensemble TA spectroscopy and single-particle TA microscopy. Additionally, we outlined common theoretical approaches to describe the generation and relaxation of these carriers, with particular emphasis on the three-temperature model (3TM) and its spatio-temporal extension (I3TM). We have also introduced the further step required to compare numerical simulations with TA spectroscopy measurements, that is, the description of the photothermal optical nonlinearities driven by high-energy electrons. This provides a comprehensive picture of the experimental and computational tools needed to investigate the photoinduced ultrafast optical modulation governed by hot carriers in plasmonic nanostructures. We finally presented examples of our contribution to this field, by reporting the most relevant works where we successfully combined ultrafast TA experiments and (I)3TM semiclassical modelling of hot electrons and related optical nonlinearities in various nanostructure configurations.

Our approach, systematically comparing experiments and simulations, has allowed us to demonstrate that the 3TM, originally introduced for thin Au films [175], can be applied to diverse plasmonic nanostructures, from nanoantennas to metasurfaces. In fact, the model neglects some important processes that occur in the first tens of femtoseconds (such as the dephasing of the plasmon) and does not distinguish hot carriers based on their specific generation process (i.e. via either interband or intraband absorption, or instead mediated by plasmonic resonances). Moreover, it considers a single time constant for the relaxation of nonthermal carriers, regardless of their energy. Nevertheless, despite such limitations, when combined with semiclassical modelling of the metal interband transitions in a suitable multiscale algorithm, the 3TM provides a remarkably good agreement with TA experiments over a broad spectral range (from the visible to the near infrared), and for a variety of sample configurations [224], including materials beyond plasmonics, such as semiconductors [185–187]. The key aspect when applying the 3TM to the modelling of TA experiments is the connection between the electron energetic degrees of freedom (i.e. the energy density of nonthermal carriers, and the temperature of thermalized ones) and the modulation of the occupation probability in the conduction band, which is substantially different for the two (i.e. non-thermal and thermal) populations of hot electrons. Moreover, the 3TM is capable of capturing and rationalizing even subtle temporal and spectral features observed in TA measurements. As an example, it gives reason of the sub-picosecond sign change observed in the TA microscopy of single Au nanoantennas as an interplay between interband transition modulations due to nonthermal or thermal carriers (the latter being generated from the former via e-e scattering).

Thanks to its computational and conceptual simplicity, the 3TM can be easily extended to the cases where generation of carriers is spatially inhomogeneous, which is the most typical situation for plasmonic nanostructures beyond the quasi-static limit (i.e. featuring sizes larger than ∼100 nm). In this framework, we have theoretically predicted and experimentally demonstrated the crucial role of the spatio-temporal dynamics of hot electrons at the nanoscale, causing a transient breaking of the nanostructure optical symmetry. This effect, almost overlooked until very recently, has been exploited to conceive a novel high-speed all-optical modulation scheme of diffraction in plasmonic metagratings. The same concept was also employed to design and experimentally demonstrate the ultrafast all-optical modulation of light polarization, employing a plasmonic metasurface made of Au nanocrosses. Thanks to the high electron thermal conductivity of noble metals, the original optical symmetry of the structure can be restored in ∼1–2 ps (depending on the specific geometry and size of the meta-atom), thus providing a full recovery of the optical signal with unprecedented speed.

These results suggest a renewed scenario for the exploitation of hot carriers in plasmonic nanostructures. The study of the ultrafast dynamics of hot electrons by TA spectroscopy was born with the original purpose of investigating the interaction phenomena between radiation and matter in metallic nanomaterials. However, the potential of TA in ultrafast plasmonics is nowadays evolving towards new directions. The possibility of exploiting the giant optical nonlinearity of noble metals, combined with the peculiar resonant properties of nanostructures, is leading to the development of new functionalities from plasmonic metasurfaces. In this framework, an accurate yet agile multiscale approach like the I3TM to model carrier generation, diffusion and relaxation, together with the subsequent transient permittivity modulation at the nanoscale, could set the stage for the design of hot-electron-based ultrafast nanophotonic devices. Moreover, the TA microscopy and I3TM analysis of ultrafast hot-carrier spatial transients is envisaged to find intriguing applications in photocatalysis. Indeed, the I3TM could introduce a so far undisclosed ingredient in this context that is the study of configurational degrees of freedom to finely tailor at the nanoscale the photogeneration of high-energy electrons in the spatial and temporal domains. This in turn can potentially lead to the development of a novel class of flat-optics antenna-reactor photocatalysts, with enhanced thermal and nonthermal performances.
